# Individual variations of the superior petrosal vein complex and their microsurgical relevance in 50 cases of trigeminal microvascular decompression

**DOI:** 10.1007/s00701-019-04109-7

**Published:** 2019-11-26

**Authors:** Mohammed Basamh, Nico Sinning, Uwe Kehler

**Affiliations:** 1 Department of Neurosurgery, Asklepios Klinik Altona, Paul-Ehrlich Strasse 1, 22763 Hamburg, Germany; 2 Division of Neurosurgery, King Abdul-Aziz University Hospital, Jeddah, Saudi Arabia

**Keywords:** Superior petrosal vein, Microvascular decompression, Cerebellopontine angle, Trigeminal neuralgia, Surgical anatomy, Anatomy

## Abstract

**Background:**

We investigated the understudied anatomical variations of the superior petrosal vein (SPV) complex (SPVC), which may play some role in dictating the individual complication risk following SPVC injury.

**Methods:**

Microvascular decompressions of the trigeminal nerve between September 2012 and July 2016. All operations utilized an SPVC preserving technique. Preoperative balanced fast field echo (bFFE) magnetic resonance imaging, or equivalent sequences, and operative videos were studied for individual SPVC anatomical features.

**Results:**

Applied imaging and operative SPVC anatomy were described for fifty patients (mean age, 67.18 years; female sex and right-sided operations, 58% each). An SPVC component was sacrificed intentionally in 6 and unintentionally in only 7 cases. Twenty-nine different individual variations were observed; 80% of SPVCs had either 2 SPVs with 3 or 1 SPV with 2, 3, or 4 direct tributaries. Most SPVCs had 1 SPV (64%) and 2 SPVs (32%). The SPV drainage point into the superior petrosal sinus was predominantly between the internal auditory meatus and Meckel cave (85.7% of cases). The vein of the cerebellopontine fissure was the most frequent direct tributary (86%), followed by the pontotrigeminal vein in 80% of SPVCs. Petrosal-galenic anastomosis was detected in at least 38% of cases. At least 1 SPV in 54% of the cases and at least 1 direct tributary in 90% disturbed the operative field. The tributaries were more commonly sacrificed.

**Conclusions:**

The extensive anatomical variation of SPVC is depicted. Most SPVCs fall into 4 common general configurations and can usually be preserved. BFFE or equivalent sequences remarkably facilitated the intraoperative understanding of the individual SPVC in most cases.

## Introduction

The superior petrosal vein (SPV) complex is a major draining system in the posterior fossa and a consistent landmark within the cerebellopontine angle (CPA) [18, 26]. Injury of a part of the SPV complex (SPVC) has been frequently reported in large case series, with an incidence ranging from 55 to 84% of cases [16, 25, 34]. However, serious complications range from 0.01 to 7% of cases [1, 9, 13, 16, 17, 23, 24, 27, 28], while minor events are probably common but underreported [5]. Lesions on magnetic resonance imaging (MRI) may be detected even in asymptomatic patients [30]. Identifying patients who are at risk of developing major complications a priori is currently impossible, because anatomical features rendering patients prone to such adverse events are unknown.

The SPVC has 1 to 2 and rarely 3 SPVs, and up to 5 possible major tributaries: the vein of the cerebellopontine fissure (v.CPF), vein of the middle cerebellar peduncle (v.MCP), pontotrigeminal vein (PTv), transverse pontine vein (TPv), and the anterior lateral marginal vein (ALMv) [18, 19, 26, 33]. The types of SPV draining into the superior petrosal sinus (SPS) have been classified [31]. An operative study advocated classification of the tributaries according to their relation to the trigeminal nerve (TGN) and a cadaveric study classified them according to their draining areas [8, 19]. The petrosal area draining veins group drains the lateral pons and medulla, fourth ventricle, and petrosal fissure. The main vein of this group is the v.CPF which forms at the suprafloccular cistern by merger of several veins and courses superiolaterally on the cerebellopontine fissure to join the SPV. The v.MCP originates above the olive to run on the middle cerebellar peduncle draining into an SPV or a v.CPF. The PTv is the major vein of the posterior mesencephalic group which drains the posterior midbrain and runs at the lateral midbrain border then within the cerebellomesencephalic fissure. The TPv drains the anterior pontomesencephalic area (anterior midbrain and upper pons) and courses transversely on the anterior pontine surface to join the SPV. The small tentorial group drains the lateral cerebellar surface facing the tentorium via the ALMv which runs over the lateral cerebellar edge towards the SPV or into one of its tributaries [18, 19, 26]. Individual variations of the SPVC and their operative implications have not yet been studied [6].

Fully refocused steady-state gradient echo MRI sequences as balanced fast field echo (bFFE), fast imaging employing steady-state acquisition (FIESTA), or three-dimensional constructive interference in steady state (3D CISS) are used routinely for the preoperative assessment in microvascular decompression (MVD) of the TGN because of its precision for evaluating neurovascular structures in the CPA cistern [3, 4, 7, 29, 35]. We collectively referred to these sequences as bFFE. Furthermore, the retrosigmoid approach in MVD of the TGN offers an ideal opportunity to study an undistorted SPVC anatomy. We aimed to evaluate the individual anatomical variations of the SPVC by examining preoperative bFFE and intraoperative findings.

## Methods

### Nomenclature

**Fig. 1:**
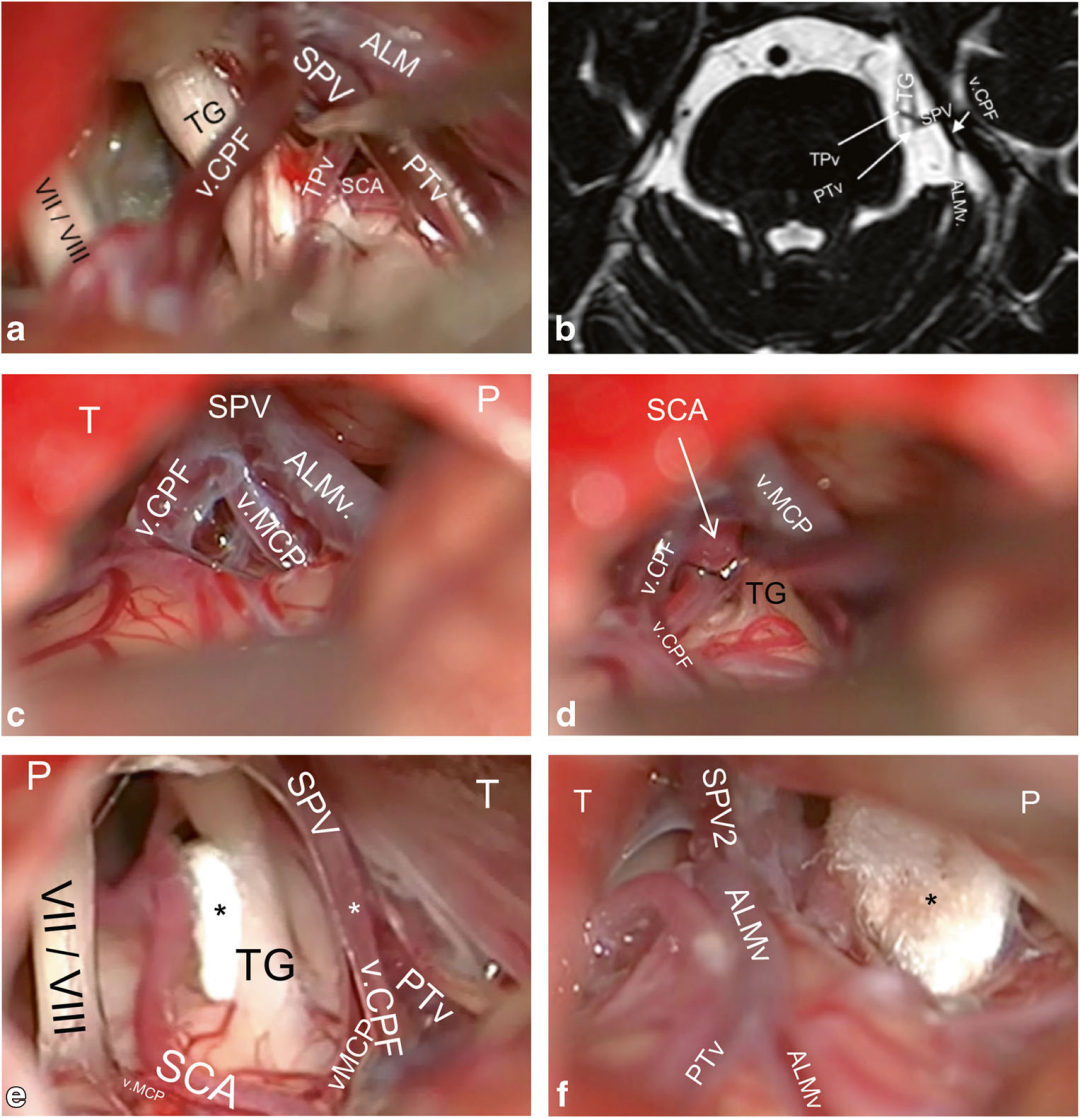
**a** A case of a left-sided MVD in a patient with an SPVC configuration of 1 SPV and 4 tributaries (1.4.) The SPV is formed by the confluence of the PTv and TPv. Note that the v.CPF and the ALMv join later at a more proximal point of the SPV. The v.CPF comes from the suprafloccular area at the cerebellopontine fissure. The flocculus is not exposed here, but the VII/VIII group indicates its location. The v.CPF has a lateral relation to the VII/VIII. The PTv comes into the operative field from a rostral direction. The TPv merges into our view from the medial and ventral aspect of the pons. **b** An axial bFFE slice from the same patient in **a** showing all 4 tributaries of the SPV intraoperatively. The PTv (only the very proximal tip is seen on this slice) and the TPv appear here, forming the SPV. The v.CPF and ALMv drain into the SPV more proximally. Interestingly, MRI also detected the TPv traversing through the TG rootlets. **c** A case of 3 huge direct tributaries on the right side obscuring the operative field completely. The SPV is very short and is barely seen. All 3 veins unite at one point to form the SPV. Note that the relationship between the veins usually could not be determined at their proximal cisternal parts. The bFFE reveals a second SPV formed by a PTv. Nevertheless, it could not be seen intraoperatively because of its medial course and field disturbance by the huge SPVC. **d** The same case as in **c**. After retracting the cerebellar edge, the distal parts of the tributaries on the pontine surface as well as their relations could be exposed. The v.CPF courses typically lateral to the v.MCP. **e** In this example of a left-sided CPA, the SPVC is causing less interference with the surgical corridor. The v.MCP is medial to the v.CPF and courses medially to VII/VIII. The SPV ends at a point between the TG and VII/VIII (drainage type II), although the angle of view is not ideal for this purpose. **f** Another example of a right CPA showing the PTv as an indirect tributary, which drains into ALMv at the pontine surface. This patient had 2 SPVs but only one of them appears here. ALMv anterior lateral marginal vein, bFFE balanced fast field echo, CPA cerebellopontine angle, MVD microvascular decompression, P petrous bone, PTv pontotrigeminal vein, SPV superior petrosal vein, SPVC superior petrosal vein complex, SPV2 second SPV, T tentorium, TG trigeminal nerve, TPv transverse pontine vein, v.CPF vein of cerebellopontine fissure, v.MCP vein of the middle cerebellar peduncle, VII/VIII seventh and eighth cranial nerves, asterisk Teflon sponge

We defined the SPVC as the SPV(s) and all of its direct tributaries [19, 31]. The SPV was defined as the proximal end(s) of the SPVC extending from the SPS to the point where the first 2 direct tributaries join or as a continuation of a single direct tributary (Fig. 1a, b) [26]. We used the term direct tributary for any vein that drained into an SPV within the CPA cistern (Fig. 1c–e). When 2 tributaries joined at the level of the cerebellar or pontine surface, the direct vein was considered the vein whose continuation drains into an SPV (Fig. 1f). Otherwise, we considered it an indirect tributary draining into a direct one.

### Patient population

We retrospectively reviewed patients’ charts, preoperative MRI scans, and operative videos between September 2012 and July 2016 in a major community hospital in Hamburg, Germany. We included all patients who underwent operation for MVD. We excluded patients who had an inadequate bFFE study, inadequate intraoperative video recording, reoperation, or an operation other than MVD of the TGN.

### Operative technique

We followed the Jannetta approach for MVD of the TGN [14, 21]. All operations were performed by the senior author (UK). Saving the SPVC was attempted in all cases by opening of the supracerebellar and then CPA cisterns with generous cerebrospinal fluid drainage, releasing the arachnoid membranes around the facial and vestibulocochlear nerves (VII/VIII) to avoid adverse stretching during the procedure, careful skeletonizing of all SPVC components that are encountered in the operative field and finally working through the corridors between the SPVC tributaries to mobilize the offending vessel and decompress the TGN (Fig. 1a, c, e, f).

### Data collection

**Fig. 2:**
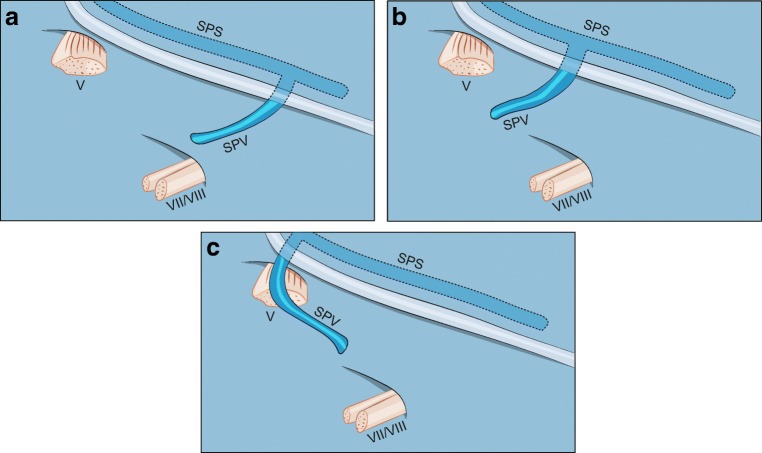
Type of SPV drainage into the SPS according to Tanriover et al. [31] **a** Type I drainage of the SPV into the SPS. **b** Type II drainage. **c** Type III drainage. SPS superior petrosal sinus, SPV superior petrosal vein, V trigeminal nerve, VII/VIII seventh and eighth cranial nerves

We reviewed patients’ age, sex, and side of the operation. For each patient, the preoperative bFFE was studied on the surgical side for the number of SPVs, number of direct tributaries in relation to the number of SPVs (for simplification this was labeled as “configuration”), individual anatomical variation of each SPVC (detailed vein combination of SPVs with their exact direct tributaries), and type of drainage into the SPS according to Tanriover et al. [31]. Type I represents a lateral, Type II an intermediate and Type III a medial pattern of drainage (Fig. 2). Whether any part of the SPVC disturbed the operative field, the incidence of intraoperative SPVC injury and whether the SPVC was compressing the TGN were documented.

Only the operative findings were considered whenever bFFE findings were discrepant. The presence of a petrosal-galenic anastomosis was assessed exclusively utilizing bFFE.

### Outcome measures

The main outcome of this study was to describe the individual anatomical variations of the SPVC as well as its relation to the operative field.

### Statistical analysis

All analyses were conducted using RStudio, version 1.1.338 (Integrated Development for R. RStudio, Inc.). Descriptive statistics of patients’ characteristics, technical characteristics of MRI, and anatomical features were collected. Analytical statistics were used to assess the association between several anatomical variables.

## Results

Fifty of 55 patients met the inclusion criteria (age range, 34-86 years; mean, 67.18 years). Women and a right-sided operation each accounted for 58% of the sample. There was no significant difference in the distribution of sex or operative side. Two-thirds of patients had MRI images from other institutions, resulting in wide variability in technical MRI specifications (Table 1).

**Table 1:** Selected patients and magnetic resonance imaging characteristics

	Range/frequency	Mean	Test and significance
Patient characteristic			
Age (years)	34–86	67.18	-
Sex			
Male	21 (42%)	-	χ^2^ = 1.28; *p* = 0.2579
Female	29 (58%)		
Side			
Right	29 (58%)	-	χ^2^ = 1.28; *p* = 0.2579
Left	21 (42%)		
MRI characteristic			
Thickness (mm)	0.4–1.5	0.99	-
Distance (mm)^1^	0.4-0.7	0.54	-
Pixel bandwidth^2^ (Hz/pixel)	130–462	339	-
Tesla^3^			
1.5	22 (44.9%)		-
3	27 (55.1%)		
Sequence^4^			
bFFE MRI	28 (56%)		
3D CISS MRI	21 (42%)		
FIESTA MRI	1 (2%)		

*bFFE* balanced fast field echo, *FIESTA* fast imaging employing steady-state acquisition, *MRI* magnetic resonance imaging, *3D CISS* three-dimensional constructive interference in steady state

^1^Data were missing in 21 of 50 cases (42%). The mean was calculated based on the available data only

^2^Missing data in 2 cases (4%). The mean was calculated based on the available data only

^3^Data were missing in 1 case (2%). The percentages shown are based on the available data only

^4^These are similar types of fully refocused steady-state gradient echo MRI sequences from different vendors, which are named differently by each vendor

### SPVC anatomy in bFFE and intraoperatively

#### Main SPV(s)

**Fig. 3:**
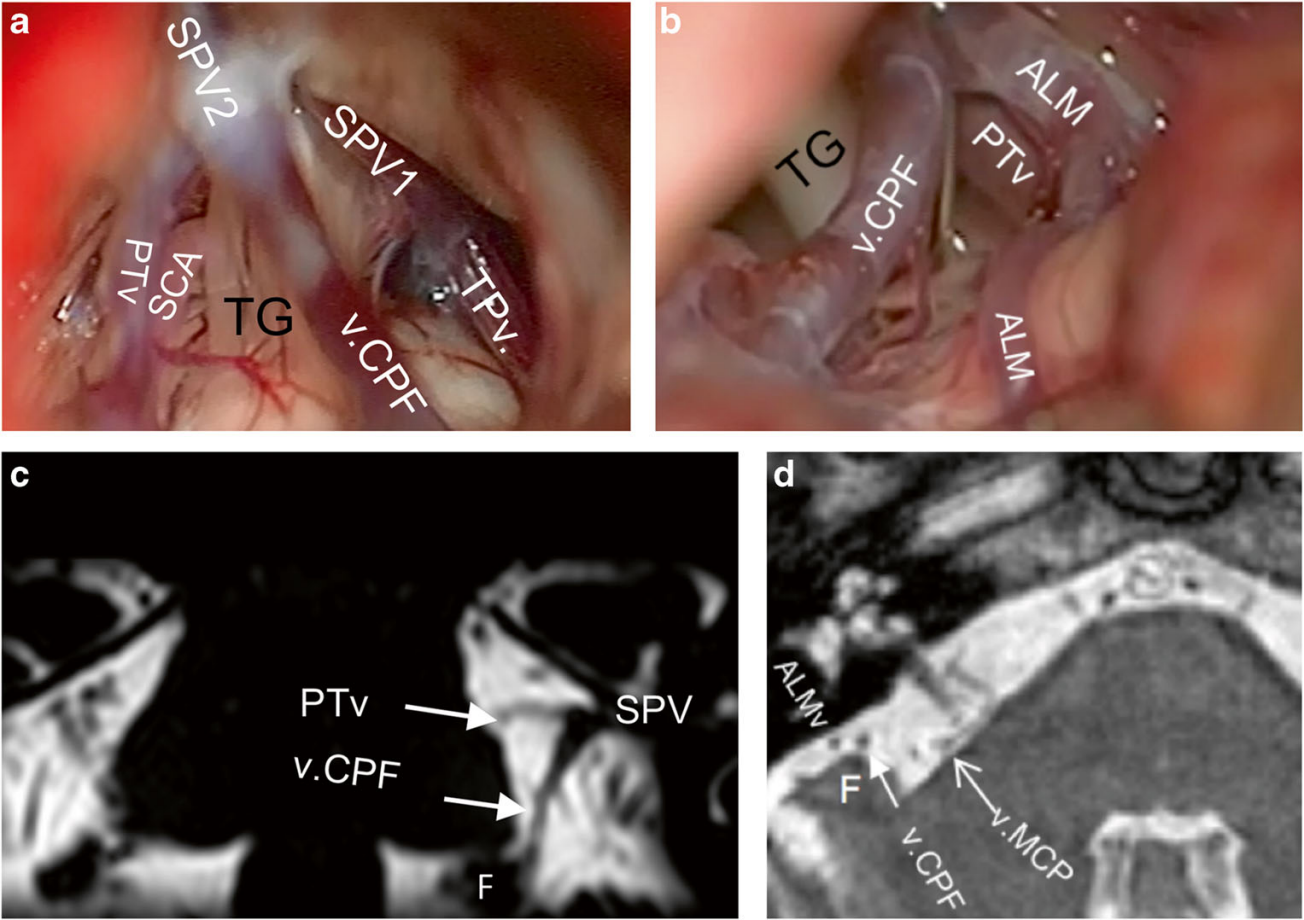
**a** Right-sided CPA showing 2 SPVs and a total of 3 direct tributaries. There is an overall moderate disturbance of the operative field. **b** Same case presented in Fig. 1a. The distal part of ALMv courses over the anterior edge of the cerebellar surface. It is the most lateral tributary of the SPV. **c** A coronal bFFE image showing the left v.CPF at its origin at the suprafloccular cistern, ascending over the cerebellopontine fissure toward the SPV. The PTv here has a proximal horizontal part and could be mistaken in this image for the TPv. However, scrolling through the images revealed a typical course of the PTv. **d** An axial cut from aright CPA showing the v.MCP running over the middle cerebellar peduncle. The v.CPF is above the flocculus. ALMv anterior lateral marginal vein, bFFE balanced fast field echo, CPA cerebellopontine angle, F flocculus, PTv pontotrigeminal vein, SCA superior cerebellar artery, SPV superior petrosal vein, SPV1 first superior petrosal vein, SPV2 second superior petrosal vein, TG trigeminal nerve, TPv transverse pontine vein, v.CPF vein of cerebellopontine fissure, v.MCP vein of the middle cerebellar peduncle

The SPV(s) could be identified at its most proximal course before it drained into the SPS at any point of its known course at the edge between the tentorium and petrous bone (Fig. 2). The length of the SPV varied; it was usually observed as a short segment (Figs. 1a, b and 3a), but it was sometimes too short to be seen intraoperatively (Fig. 3b).

The exact vein of each tributary was identified in bFFE by tracking its course from the SPV(s) to its most distally seen point.

#### Vein of the cerebellopontine fissure

In bFFE, the vein of the cerebellopontine fissure originated in the suprafloccular cistern, just above the flocculus. It ran through the cerebellopontine fissure and merged into the CPA cisterns toward the SPV (Fig. 3c, d). The v.CPF was commonly seen directly upon exposing the upper CPA angle with the brain spatula. It could be seen at its origin lateral to the VII/VIII (Fig. 1a).

#### Vein of the middle cerebellar peduncle

If present, the vein of the middle cerebellar peduncle had a parallel but more medial course to the v.CPF. It merged from the caudal end of the operative field, medial to the VII/VIII (Fig. 1e). In bFFE, it was easier to identify the distal part of v.MCP running on the surface of the middle cerebellar peduncle (Fig. 3d) before it traveled further either to drain into the v.CPF on the cerebellopontine fissure or to continue intracisternally to join an SPV.

#### Pontotrigeminal vein

**Fig. 4:**
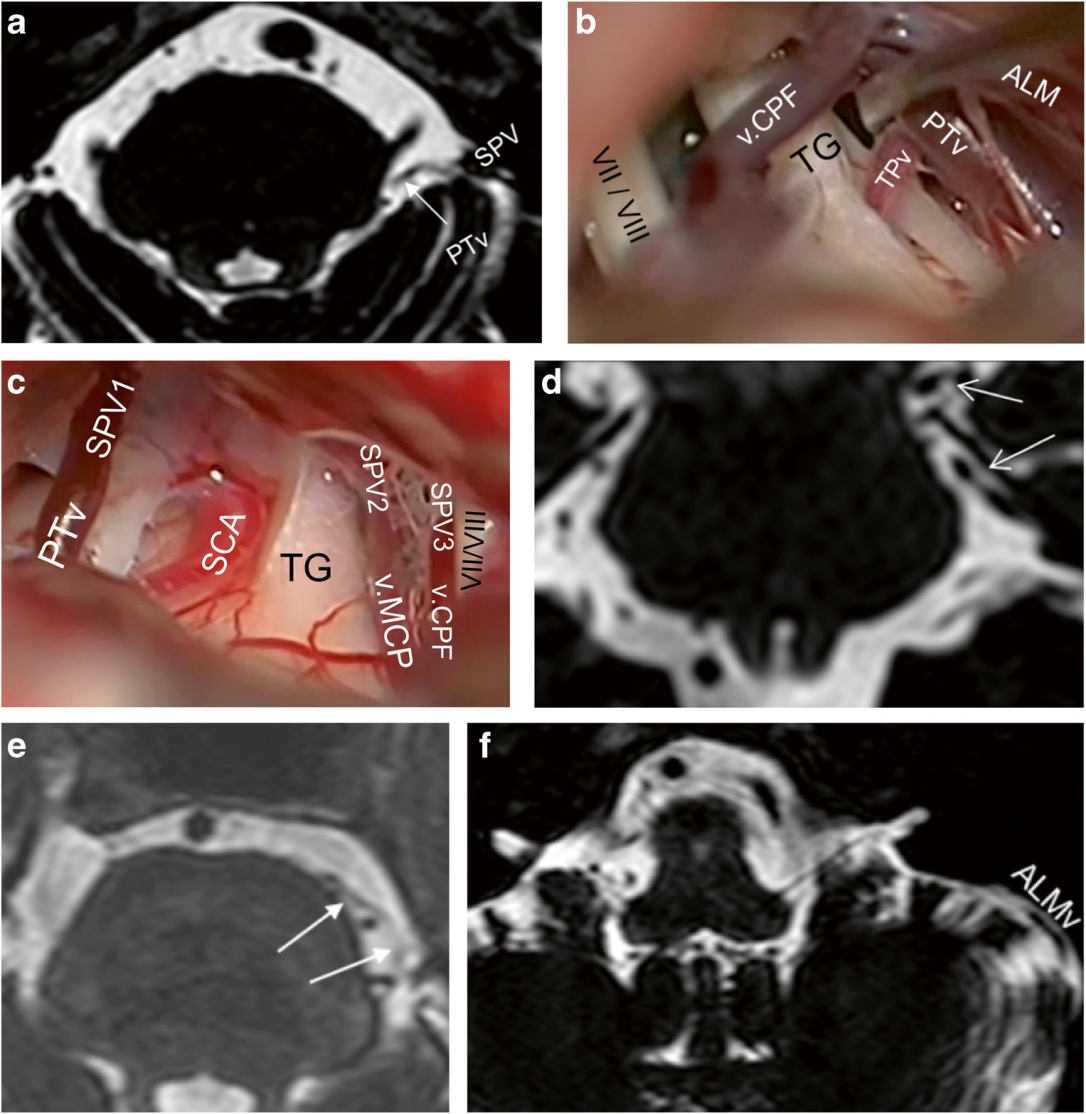
**a** An axial bFFE cut showing the left PTv leaving the lateral pontine sulcus to drain into the SPV. **b** A closer view from the same case of Fig. 1a showing the TPv traversing the trigeminal nerve. **c** Right-sided CPA showing 3 SPVs with 3 direct tributaries. This SPVC causes only minimal disturbance of the operative field. **d** A coronal bFFE cut showing the left PTv (lower arrow) and basal vein of Rosenthal (upper arrow) pointing to each other. Further scrolling of the images showed that they anastomose. **e** An axial bFFE cut showing the left TPv with its horizontal course originating at the ventral pons. **f** An axial bFFE cut at a lower level of the posterior fossa showing the left ALMv running over the anterior lateral edge of the cerebellar surface. ALMv anterior lateral marginal vein, bFFE balanced fast field echo, CPA cerebellopontine angle, PTv pontotrigeminal vein, SPV superior petrosal vein, SPVC superior petrosal vein complex, TG trigeminal nerve, TPv transverse pontine vein, v.CPF vein of cerebellopontine fissure, v.MCP vein of the middle cerebellar peduncle, VII/VIII seventh and eighth cranial nerves

The PTv was always identified in bFFE while descending along the lateral pontine sulcus (Fig. 4a); thus, it was the only tributary to emerge from the rostral side of the operative field (Figs. 1a, e and 4b, c). Its course was always superoposterior to inferoanterior.

#### Petrosal-galenic anastomosis via the PTv

The rostral course of the PTv could be tracked using bFFE in 19 cases (38%) until the ambient cistern, where it either continued with the basal vein of Rosenthal in 9 cases or into the quadrigeminal cistern draining into the great vein of Galen in 10 cases (Fig. 4d, Table 2). There was no interconnection in 9 cases (18%). The PTv was absent in 8 (16%), present but not draining into an SPV in 1 case (2%), and the interconnection could not be assessed in the remaining 13 cases (26%). Twelve of the later 13 cases had inadequate MRI acquisition rostrally (mostly the cuts did not reach the level of the midbrain), and artifacts disturbed the upper cuts in 1 case. Because this interconnection was not possible to assess intraoperatively, the certainty of this MRI observation was evaluated; it was felt to be certain in 41 cases (82%) and unsure in the remaining 9 (18%). The unsure findings involved different observations of this interconnectivity, without a certain tendency toward one of them.

**Table 2:** A tabular description of the petrosal-galenic anastomosis as seen in balanced fast field echo magnetic resonance imaging

Petrosal-galenic anastomosis	Prevelance (*n* = 50)
Present	38% (19)
Absent	18% (9)
Non-assessable	26% (13)
Inadequate bFFE^1^	13 of 13
PTv absent	16% (8)
PTv not part of the SPVC	2% (1)

*bFFE* balanced fast field echo, *PTv* pontotrigeminal vein, *SPVC* superior petrosal vein complex

^1^The number of cases with non-assessable petrosal-galenic anastomosis due to technical shortcomings of the bFFE magnetic resonance imaging examinations

#### Transverse pontine vein

The TPv was the only vein to merge from the medial and anterior aspects of the pons (Figs. 1a, b, 3a, and 4b). It had a clear horizontal course in bFFE (Fig. 4e).

#### Anterior lateral marginal vein

The ALMv always had a distinct course along the anterolateral edge of the cerebellar surface facing the tentorium (Fig. 4f). Sometimes it could be intraoperatively mistaken for the v.CPF. It sloped rostrally but slighter than the v.CPV and v.MCP, and as opposed to them, it had a lateral to medial course. It was always the most lateral vein, and if present, it was the first to be seen when advancing the brain spatula superficially before exposing the cerebellopontine fissure and the CPA cistern (Figs. 1f and 3b). Although it was usually small, it had a larger size in some cases (Fig. 1c).

### Individual variations of the SPVC

**Fig. 5:**
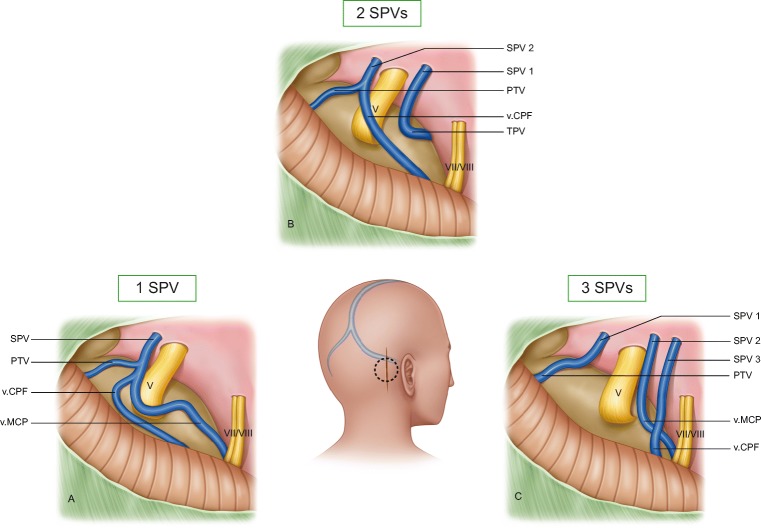
The 3 main patterns of an SPVC. **a** An SPVC with a single SPV. The general configuration here is 1 SPV with 3 tributaries, which was the most common SPVC configuration in the whole sample. **b** An SPVC with 2 SPVs. The configuration here is 2 SPVs with 3 tributaries, which was along with the configuration of 1 SPV with 2 tributaries, the second most common in the sample. **c** An SPVC with 3 SPVs. ALMv anterior lateral marginal vein, PTv pontotrigeminal vein, SPS superior petrosal sinus, SPV superior petrosal vein, , SPVC superior petrosal vein complex, SPV1 first superior petrosal vein, SPV2 second superior petrosal vein, SPV3 third superior petrosal vein, TPv transverse pontine vein, v.CPF vein of cerebellopontine fissure, v.MCP vein of the middle cerebellar peduncle, V trigeminal nerve, VII/VIII seventh and eighth cranial nerves

Seventy SVPs were seen in 50 patients. One SPV was observed 32 times (64%), 2 were observed 16 times (32%), and 3 SPVs twice (4%) (Fig. 5).

Nearly half of the cases had 3 and about a quarter had 2 or 4 tributaries each (Table 3). One or 5 tributaries was rarely present. Among 153 direct tributaries, the v.CPF and PTv were present in most cases, whereas the TPv, v.MCP, and ALMv were present in only nearly half or less of them (Table 4). The observed sizes of each SPVC component varied widely*.*

**Table 3:** The prevalence of the number of tributaries in an superior petrosal vein complex and its relation to the surgical field

Number of tributaries in an SPVC	Prevalence	Presence of a vein compressing the TGN	Number of disturbing tributaries in an SPVC	Prevalence
1	1 (2%)	0	1	11 (22%)
2	12 (24%)	1*	2	21 (42%)
3	21 (42%)	7^1^	3	10 (20%)
4	13 (26%)	4	4	3 (6%)
5	2 (4%)	2*	5	0
			0	5 (10%)
Total	50 cases	14 of 50 cases	Total	50 cases

*TGN* trigeminal nerve, *SPVC* superior petrosal vein complex

^*^Each of these groups had 1 case with 2 offending veins that compressed the TGN. All other cases had only a single offending vein

^1^In 1 of these cases, only 1 SPV was the culprit vein without involvement of tributaries. This case had 2 main SPVs

**Table 4:** Prevalence of each superior petrosal vein complex tributary and its relation to the surgical field

Tributary veins	Prevalence (% from 50 cases)	Doubled tributaries (% from 50 cases)	Disturbing the operative field (% from 50 cases)	Compressing the TGN (% from 50 cases)
v.CPF	43 (86%)	0	34 (68%)	3 (6%)
PTv	40 (80%)	7 (14%)	33 (66%)	2 (4%)
v.MCP	25 (50%)	-	13 (26%)	2 (4%)
TPv	25 (50%)	1 (2%)	6 (12%)	7 (14%)
ALMv	20 (40%)	1 (2%)	11 (22%)	-
Total	153 tributaries	9 veins in 8 cases		14 veins in 13 cases^1^

*ALMv* anterior lateral marginal vein, *PTv* pontotrigeminal vein, *SPVC* superior petrosal vein complex, *TPv* transverse pontine vein, *v.CPF* vein of the cerebellopontine fissure, *v.MCP* vein of the middle cerebellar peduncle

^1^There were 14 cases with venous compression of the trigeminal nerve with a total of 16 offensive veins. One case had 1 of 2 main SPVs as the single culprit vein and was therefore not taken into consideration in this table. Another 2 cases had 2 offensive veins; 1 of 2 SPVs and a TPV in 1 case and a v.CPF and a TPv in the other case

We detected 29 different individual variations of the SPVC in our 50 cases. There were 10 general configurations. We referred to them with a 2-digit code to simplify the presentation of our data, where the first and second digit implies the number of SPV(s) and number of direct tributaries, respectively (Table 5). Most cases (80%) fell into 1 of 4 overwhelmingly common SPVC configurations: 1.3 (24%), 1.2 (20%), 2.3, and 1.4 (18% each). Each of the remaining configurations was uncommon.

**Table 5:** Anatomical variations of superior petrosal vein complexes

Number of SPVs (number of patients)	General configuration^1^ (number of patients)		Individual variation	Number of patients
1 (32)	1.1 (1)	1.1.1	SPV: TPv	1
	1.2 (10)	1.2.1	SPV: v.CPF + PTv	5
		1.2.2	SPV: v.CPF + TPv	2
		1.2.3	SPV: v.CPF + v.MCP	1
		1.2.4	SPV: v.CPF + v.ALM	1
		1.2.5	SPV: PTv + v.MCP	1
	1.3 (12)	1.3.1	SPV 1: v.CPF + PTv + TPv	2
		1.3.2	SPV 1: v.CPF + PTv + v.MCP	3
		1.3.3	SPV 1: v.CPF + PTv + ALMv	1
		1.3.4	SPV 1: v.CPF + TPv + v.MCP	2
		1.3.5	SPV 1: PTv + TPv + ALMv	1
		1.3.6	SPV 1: PTv + v.MCP + ALMv	3
	1.4 (9)	1.4.1	SPV 1: v.CPF + PTv + TPv + v.MCP	2
		1.4.2	SPV 1: v.CPF + PTv + TPv + ALMv	5
		1.4.3	SPV 1: v.CPF + PTv + v.MCP + ALMv	2
2 (16)	2.2 (2)	2.2.1	SPV 1: v.CPF SPV 2: TPv	1
		2.2.1	SPV 1: v.CPF SPV 2: ALMv	1
	2.3 (9)	2.3.1^2^	SPV 1: PTv1 SPV 2: v.CPF + PTv2	1
		2.3.2	SPV 1: PTv SPV 2: v.CPF + v.MCP	1
		2.3.3	SPV 1: TPv SPV 2: v.CPF + PTv	4
		2.3.4	SPV 1: TPv SPV 2: v.CPF + ALMv	1
		2.3.5	SPV 1: v.MCP SPV 2: v.CPF + PTv	2
	2.4 (3)	2.4.1	SPV 1: v.CPF SPV 2: PTv + v.MCP + ALMv	1
		2.4.2	SPV 1: PTv SPV 2: v.CPF + v.MCP + ALMv	1
		2.4.3	SPV 1: TPv SPV 2: v.CPF + PTv + ALMv	1
	2.5 (2)	2.5.2	SPV 1: ALMv SPV 2: v.CPF + PTv + TPv + v.MCP	1
		2.5.2^1^	SPV 1: PTv + ALMv SPV 2: v.CPF + TPv + v.MCP	1
3 (2)	3.3 (1)	3.3.1	SPV 1: v.CPF SPV 2: PTv SPV 3: v.MCP	1
	3.4 (1)	3.4.1	SPV 1: TPv SPV 2: v.MCP SPV 3: PTv + ALMv	1

^1^A 2-digit code was designated to each configuration of the SPVC, where the first digit implied the number of SPVs and the second digit dictated the total number of the tributaries. The detailed venous combination (individual SPVC anatomical variation) that occurred under the same configuration was numbered consecutively. We referred to each exact individual variation with a 3-digit code. The third digit refers to the number of the venous combination

^2^This case had a doubled PTv, each one drained into a separate SPV. All other cases of doubled direct tributaries had both veins draining directly into the same SPV, or one of them drained into another direct tributary. For the sake of simplification, we considered these doubled veins as one

**Fig. 6:**
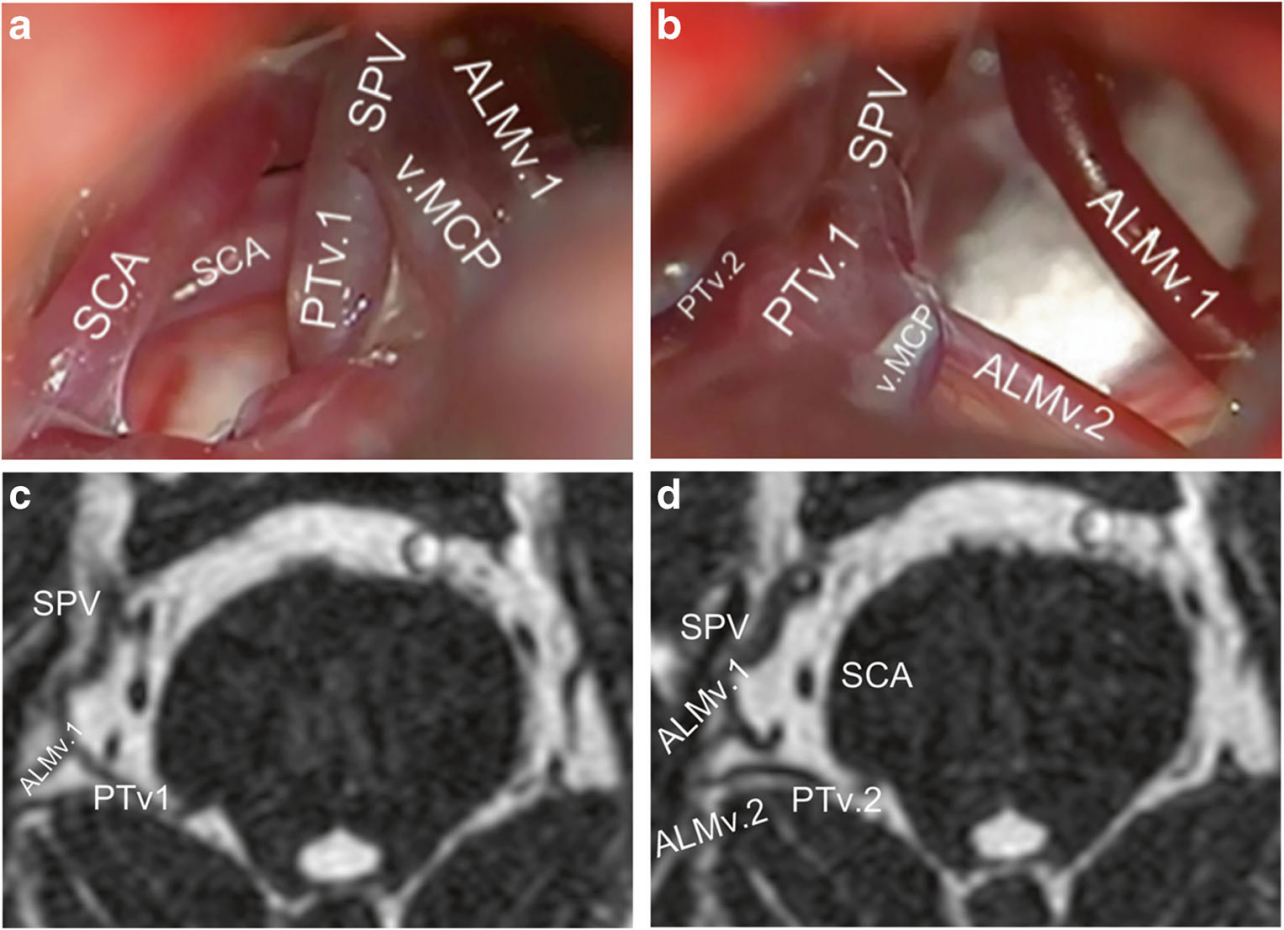
A case of right-sided CPA with 2 different doubled SPV’s tributaries. **a** An intraoperative retrosigmoid view showing the SPV composed of a PTv1 and v.MCP, and ALMv1 joined the SPV at a more proximal point. A Teflon pad appears in the background. **b** View from a slightly different angle showing a second ALMv (ALMv2) as draining into a PTv2, which did not drain into the SPV. **c**, **d** Correspondent findings in bFFE axial views. Note that the v.MCP appeared in other slices, which are not shown here. Additionally, further bFFE slices show that the PTv2 drains directly into the vein of Rosenthal in this case without being part of the SPVC. ALMv1 fist anterior lateral marginal vein, ALMv2 second anterior lateral marginal vein, bFFE balanced fast field echo, CPA cerebellopontine angle, PTv1 first pontotrigeminal vein, PTv2 second pontotrigeminal vein, SCA superior cerebellar artery, SPV superior petrosal vein, SPVC superior petrosal vein complex, v.MCP vein of the middle cerebellar peduncle

Overall, 8 patients (16%) had at least 1 doubled tributary, with 9 doubled veins in total (Fig. 6). One of those patients (2%) had 2 doubled tributaries, which were a doubled PTv and a doubled ALMv. Most doubled tributaries were direct ones. The most frequent doubled vein was the PTv (Table 4). There was no tendency for the presence of doubled tributaries toward neither the number of SPVs or any configuration (*χ*^2^ [9] = 9.18; *p* = 0.5825, Fisher exact test).

**Fig. 7:**
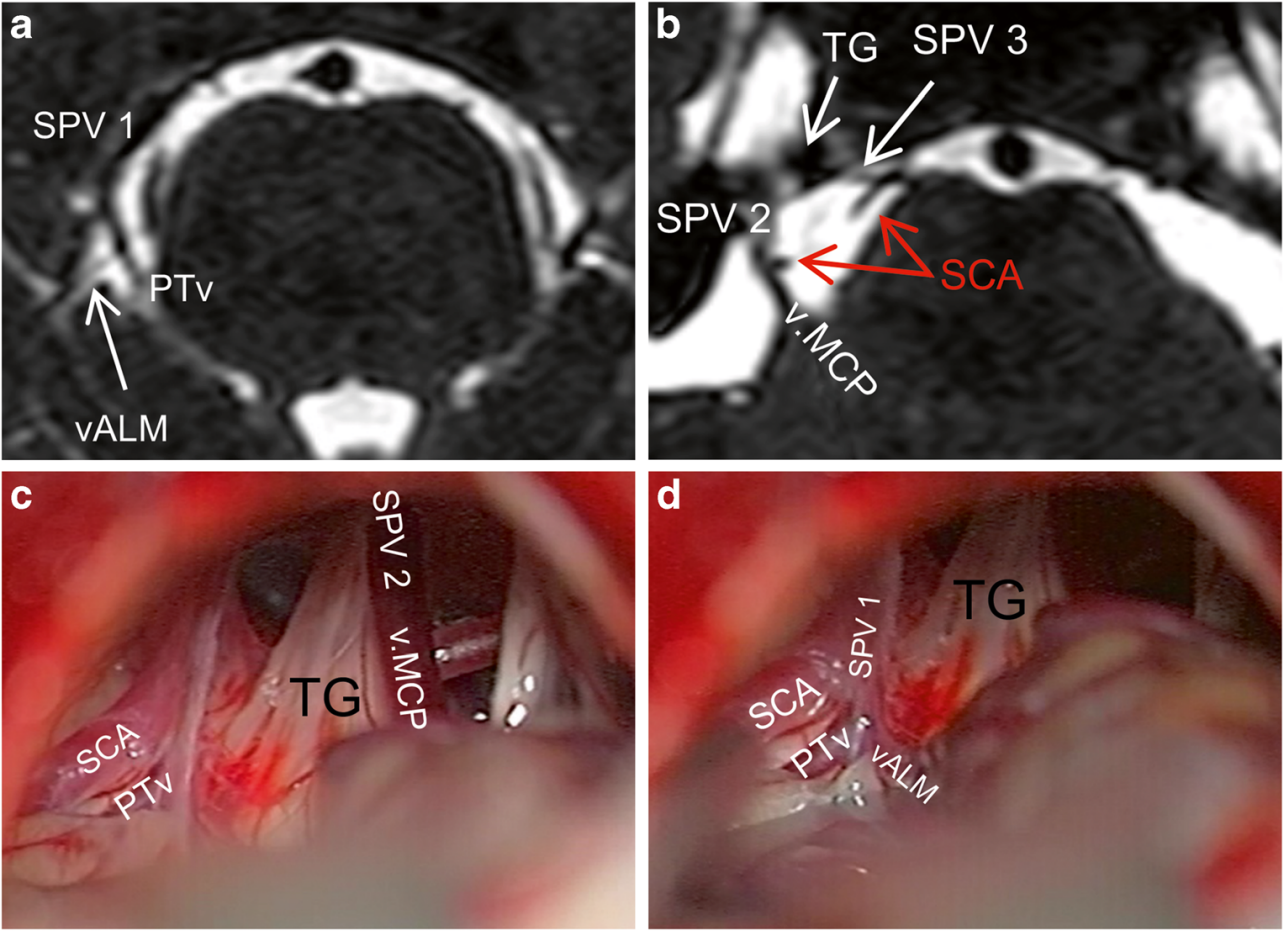
A case of right-sided CPA. **a** Axial bFFE magnetic resonance imaging showing an SPV1 composed of an ALMv and a PTv. **b** Another axial section showing an SPV 2 as the continuation of the v.MCP as well as an SPV 3 as a continuation of an TPv. **c**, **d** Corresponding intraoperative photographs of the same case. We can see here all the above described SPVC components, except the SPV 3 and its single tributary (TPv). This is because of the very medial course of the TPv and medial type III drainage of the SPV 3 into the SPS beyond the microsurgical field. ALMv anterior lateral marginal vein, bFFE balanced fast field echo, CPA cerebellopontine angle, SCA superior cerebellar artery, SPV superior petrosal vein, SPVC superior petrosal vein complex, SPV1 first SPV, SPV2 second SPV, SPV3 third SPV, TG trigeminal nerve, TPv transverse pontine vein, v.CPF vein of cerebellopontine fissure, v.MCP vein of the middle cerebellar peduncle

Of 70 SPVs, type II drainage into the SPS was predominant, occurring in 60 SPVs (85.7%) (Fig. 2). There were 2 cases in which type III drainage was located too medially beyond the surgical exposure, thus, seen in bFFE but not intraoperatively (Fig. 7).

### Interference of the SPVC with the operative field

The operative field was disturbed by 1 or more SPVs in 27 of 50 patients (54%). In SPVCs with 1 SPV, the operative field was disturbed by the single SPV in 46.9% of cases. With 2 SPVs, 1 of 2 SPVs disturbed the operative field in 7 of 16 cases (43.7%), and both SPVs disturbed the operative field in 18.7%. Both CPAs with 3 SPVs were disturbed by 2 of 3 existing SPVs (100%).

**Fig. 8:**
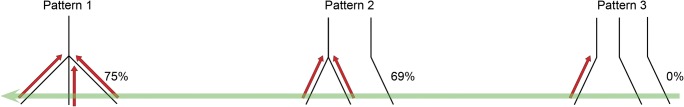
A sketch representing, from left to right, Figs. 1c, 3a, and 4c, which has 1, 2, and 3 main SPVs, respectively. Each of them had a total of 3 tributaries. The incidence of operative field disturbance by more than 1 tributary (shown as percentages) is related inversely with the number of SPVs present (as the green arrow demonstrates). The red arrows demonstrate the number of tributaries converging into an SPV. SPV superior petrosal vein

Overall, the operative field was disturbed by at least 1 tributary in 45 cases (90%); 21 cases had 2, and the rest had 1, 3, or rarely 4 disturbing tributaries (Table 3). The extent of disturbance ranged from total blockage to no disturbance of the field (Figs. 1c, 3a, 4c, and 8). The frequency of having ≥ 2 disturbing tributaries was 75% in cases with 1 SPV, 31.2% with 2 SPVs, and 0% with 3 SPVs (Fig. 8).

Direct tributaries interfered with the operative field with the same frequency of its prevalence, except for the TPv, which was the least frequent (Table 4). However, the TPv was the most common to compress the TGN in half of the 14 cases with venous compression. A main SPV in 2 cases and at least 1 tributary in 13 cases compressed the TGN.

A part of the SPVC was sacrificed in 13 of 50 cases without significant association with the disturbance of the operative field and without clinical complications. In 8 of those cases, a vein was cut intentionally to facilitate the approach. In 2 cases an SPV was severed. The most commonly severed direct tributaries were the PTv in 7 of 13 cases (53.8%), followed by TPv in 3 cases (23%) and v.CPF in 1 case.

## Discussion

### SPVC anatomy

Previous smaller cadaveric and angiographic studies found a similar prevalence of the number of SPVs, with 1 SPV as the most common [12, 19, 31, 33]. Conversely, one paper[18] reported a predominance of 2 SPVs, but this is likely attributable to their smaller sample. Operative studies are larger but lack detailed anatomical evaluation [15, 16, 22, 25, 33, 34]. A large non-English operative study found that 2 SPVs was the most prevalent [34]. Their definitions of SPVC components were unclear in the English abstract, and they could have been different from ours. For instance, we commonly observed a very short SPV, confirming a previous narrative finding [6], where the direct tributaries may be misinterpreted for main SPVs (Fig. 1c). Further, we noted that multiple SPVs always drained into the SPS at different points. If an SPV drains very medially, it is usually not seen intraoperatively (Fig. 7). Herein, bFFE helped to overcome such pitfalls.

An unspecific definition of SPVC components is frequent in the literature [1, 12, 15, 16, 21, 25, 27, 33]. We suggested thorough and practical definitions of SPVC components, and differentiated between direct and the surgically irrelevant indirect tributaries to avoid confusion. A unified nomenclature would ease communication in future studies.

The v.CPF is the most frequent tributary followed by the PTv [18, 19, 26, 33, 34]. Absence of the SPV is very rare [20].

### Individual SPVC variations

The individual SPVC variations showed huge variability, which stresses the role of preoperative evaluation. Strikingly, over three-quarters of our cases had 2 SPVs with 3 direct tributaries or a single SPV receiving 2, 3, or 4 direct tributaries regardless of the exact tributary combination.

Nearly a quarter of the patients had at least 1 doubled tributary (Fig. 6, Table 4). This most likely represents a redundancy in the venous drainage of the respective drained area. Consequently, it is probably safe to sacrifice one of these duplicates, if improvement of the surgical exposure is warranted.

The petrosal-galenic anastomosis is formed by the lateral mesencephalic or superior cerebellar vein and is well described [2, 10, 18, 19, 26]. There are few data on the frequency of this anastomosis. One study found it in 77.8% of 45 normal subjects using three-dimensional computer tomography venography (3D CTV) [22]. Other cadaveric studies described it in all cases [2, 10], the largest study of these had 52 hemispheres (only 26 brains). We found that this anastomosis is detectable in at least 38% of cases, adding to the importance of bFFE for individualized preoperative assessment (Fig. 4D, Table 2). Absence of this infra-supratentorial anastomosis may increase the risk of complications from SPVC injury.

We noticed that SPVs with uncommon (7.1%) lateral drainage into the SPS (type I) stretched substantially under minimal cerebellar retraction; thus, it was the most challenging type.

### bFFE MRI and SPVC anatomy

The bFFE helped significantly in clarifying the intraoperative venous anatomy, especially in identifying the tributaries. Nonetheless, the MRI technical specifications, and thus quality, were obviously variable in this sample (Table 1).

The bFFE sequence is used routinely to preoperatively evaluate the anatomy of cranial nerves, major arteries and neurovascular contact in the CPA [3, 7, 35]. Different MRI sequences were studied in a report of postoperative lesions [30]. However, we could not find any study of SPVC anatomy using bFFE. We have subjectively noticed a significant learning curve for a detailed understanding of the SPVC anatomy in bFFE.

### SPVC and disturbance of the operative field

The frequency of the SPV(s) interfering with the operative field was directly proportionate to the increasing number of SPVs. Overall, an SPV disturbed the field in 54% of cases. On the other hand, the number of SPVs was related inversely with the number of direct tributaries interfering with the surgical field, and this is likely because the fewer the SPVs, the more direct tributaries converge toward the same point, i.e., the SPV (Figs. 1c, 3a, 4c, and 8).

In 90% of cases, there was at least 1 direct tributary disturbing the surgical approach; in half of which there were 2 tributaries. The frequency of the culprit direct tributary veins followed their overall prevalence, except for the TPv. This could be explained by its anteromedial course, which makes it usually located away from the operative field. This emphasizes the worth of preoperative and intraoperative evaluations of the whole SPVC rather than the main SPV(s) only[11]. The TPv was the most common offending vein, similar to previous reports [8, 32].

Although SPVC components commonly block the surgical exposure, preserving it is possible in many cases by utilizing adequate cisternal drainage and careful development of intervenous corridors [8, 9, 11, 32]. This proved to be effective in the cases reviewed in this study. Dissecting the veins from the arachnoid membranes also allowed gentle retraction on the veins to widen the working corridor if needed (Fig. 1d and 4b). In only 6 cases (12%) was sacrificing an SPVC component necessary to aid the surgical exposure; in 2 cases to decompress the TGN.

The indirect tributaries were surgically irrelevant, as they never disturbed the surgical field and, therefore, were never cut.

## Limitations

The sample was retrospective with relatively small size and technical features of bFFE were heterogenous. Limited surgical corridors, such as those used for MVD operations, offer a narrow inspection of the veins and its anatomical course compared with wide exploration in cadaveric studies. Finally, the accuracy of bFFE in assessing SPVC has to be studied.

## Conclusions

We provided extensive quantitative and qualitative analyses of the SPVC anatomy with emphasis on individual variations in 50 patients. The microsurgical implications for the retrosigmoidal approach were discussed. The routinely available bFFE was valuable in improving intraoperative understanding and adding relevant information beyond the surgical field. We are currently validating our results in more CPAs and studying the accuracy of bFFE compared with the intraoperative findings. Finally, we will report a detailed analysis of the individual anatomy, including vein sizes, of all cases with SPVC injuries and explore the anatomical reasoning behind the relative low incidence of clinical complications and potentially, the individual risk assessment.

## Abbreviations

ALMv: Anterior lateral marginal vein
bFFE: Balanced fast field echo
CPA: Cerebellopontine angle
F: Flocculus
MRI: Magnetic resonance imaging
MVD: Microvascular decompression
PTv: Pontotrigeminal vein
SCA: Superior cerebellar artery
SPS: Superior petrosal sinus
SPV: Superior petrosal vein
SPVC: Superior petrosal vein complex
TG or TGN: Trigeminal nerve
TPv: Transverse pontine vein
V: Trigeminal nerve
VII/VIII: Seventh and eighth cranial nerves
v.CPF: Vein of cerebellopontine fissure
v.MCP: Vein of middle cerebellar peduncle.

## References

[1] Anichini G, Iqbal M, Rafiq N, Ironside J, Kamel M (2016). Sacrificing the superior petrosal vein during microvascular decompression. Is it safe? Learning the hard way. Case report and review of literature. Surg Neurol Int.

[2] Ardeshiri A, Ardeshiri A, Linn J, Tonn J-C, Winkler PA (2007). Microsurgical anatomy of the mesencephalic veins. J Neurosurg.

[3] Besta R (2016). MRI 3D C ISS – A novel imaging modality in diagnosing trigeminal neuralgia – a review. J Clin Diagn Res.

[4] Chavhan GB, Babyn PS, Jankharia BG, Cheng HLM, Shroff MM (2008). Steady-state MR imaging sequences: physics, classification, and clinical applications. Radiographics.

[5] Cheng L (2016). Complications after obliteration of the superior petrosal vein: are they rare or just underreported?. J Clin Neurosci.

[6] Choudhari KA (2007). Superior petrosal vein in trigeminal neuralgia. Br J Neurosurg.

[7] Docampo J, Gonzalez N, Munoz A, Bravo F, Sarroca D, Morales C (2015). Neurovascular study of the trigeminal nerve at 3 T MRI. Neuroradiol J.

[8] Dumot C, Sindou M (2015). Trigeminal neuralgia due to neurovascular conflicts from venous origin: an anatomical-surgical study (consecutive series of 124 operated cases). Acta Neurochir.

[9] Dumot C, Sindou M (2018). Veins of the cerebellopontine angle and specific complications of sacrifice, with special emphasis on microvascular decompression surgery. a review. World Neurosurg.

[10] Ebner FH, Roser F, Shiozawa T, Ruetschlin S, Kirschniak A, Koerbel A, Tatagiba M (2009). Petrosal vein occlusion in cerebello-pontine angle tumour surgery: an anatomical study of alternative draining pathways. Eur J Surg Oncol.

[11] Feng B, Zheng X, Wang X, Wang X, Ying T, Li S (2015). Management of different kinds of veins during microvascular decompression for trigeminal neuralgia: technique notes. Neurol Res.

[12] Gharabaghi A, Koerbel A, Löwenheim H, Kaminsky J, Samii M, Tatagiba M (2006). The impact of petrosal vein preservation on postoperative auditory function in surgery of petrous apex meningiomas. Neurosurgery.

[13] Inamasu J, Shiobara R, Kawase T, Kanzaki J (2002). Haemorrhagic venous infarction following the posterior petrosal approach for acoustic neurinoma surgery: a report of two cases. Eur Arch Oto-Rhino-Laryngol.

[14] Jannetta PJ, McLaughlin MR, Casey KF (2005). Technique of microvascular decompression. Technical note. Neurosurg Focus.

[15] Koerbel A, Gharabaghi A, Safavi-Abbasi S, Samii A, Ebner FH, Samii M, Tatagiba M (2009). Venous complications following petrosal vein sectioning in surgery of petrous apex meningiomas. Eur J Surg Oncol.

[16] Liebelt BD, Barber SM, Desai VR, Harper R, Zhang J, Parrish R, Baskin DS, Trask T, Britz GW (2017). Superior petrosal vein sacrifice during microvascular decompression: perioperative complication rates and comparison with venous preservation. World Neurosurg.

[17] Masuoka J, Matsushima T, Hikita T, Inoue E (2009). Cerebellar swelling after sacrifice of the superior petrosal vein during microvascular decompression for trigeminal neuralgia. J Clin Neurosci.

[18] Matsushima T, Rhoton AL, de Oliveira E, Peace D (1983). Microsurgical anatomy of the veins of the posterior fossa. J Neurosurg.

[19] Matsushima K, Matsushima T, Kuga Y, Kodama Y, Inoue K, Ohnishi H, Rhoton AL (2014). Classification of the superior petrosal veins and sinus based on drainage pattern. Neurosurgery.

[20] Matsushima K, Carvalhal Ribas E, Kiyosue H, Komune N, Miki K, Rhoton A (2015). Absence of the superior petrosal veins and sinus: surgical considerations. Surg Neurol Int.

[21] McLaughlin MR, Jannetta PJ, Clyde BL, Subach BR, Comey CH, Resnick DK (1999). Microvascular decompression of cranial nerves: lessons learned after 4400 operations. J Neurosurg.

[22] Mizutani K, Toda M, Yoshida K (2016). The analysis of the petrosal vein to prevent venous complications during the anterior transpetrosal approach in the resection of petroclival meningioma. World Neurosurg.

[23] Nakase H, Shin Y, Nakagawa I, Kimura R, Sakaki T (2005). Clinical features of postoperative cerebral venous infarction. Acta Neurochir.

[24] Narayan V, Savardekar AR, Patra DP, Mohammed N, Thakur JD, Riaz M, Nanda A (2018). Safety profile of superior petrosal vein (the vein of Dandy) sacrifice in neurosurgical procedures: a systematic review. Neurosurg Focus.

[25] Pathmanaban ON, O’Brien F, Al-Tamimi YZ, Hammerbeck-Ward CL, Rutherford SA, King AT (2017). Safety of superior petrosal vein sacrifice during microvascular decompression of the trigeminal nerve. World Neurosurg.

[26] Rhoton ALJ (2000) The posterior fossa veins. In: Rhoton AL (ed) Posterior Cranial Fossa Microsurg. Anat. Surg. Approaches. Williams & Wilkins Co, pp S69–S92

[27] Ryu H, Yamamoto S, Sugiyama K, Yokota N, Tanaka T (1999). Neurovascular decompression for trigeminal in elderly patients. Neural Med Chir.

[28] Singh D, Jagetia A, Sinha S (2006). Brain stem infarction: a complication of microvascular decompression for trigeminal neuralgia. Neurol India.

[29] Subha M, Arvind M (2019). Role of magnetic resonance imaging in evaluation of trigeminal neuralgia with its anatomical correlation. Biomed Pharmacol J.

[30] Takase Y, Kawashima M, Matsushima T, Masuoka J, Nakahara Y, Inoue K (2014). of Cerebellar damage after microvascular decompression surgery for trigeminal neuralgia: special reference to the effects of superior petrosal vein sacrifice and cerebellar compression with a spatula. JSM Neurosurg Spine.

[31] Tanriover N, Abe H, Rhoton AL, Kawashima M, Sanus GZ, Akar Z (2007). Microsurgical anatomy of the superior petrosal venous complex: new classifications and implications for subtemporal transtentorial and retrosigmoid suprameatal approaches. J Neurosurg.

[32] Toda H, Iwasaki K, Yoshimoto N, Miki Y, Hashikata H, Goto M, Nishida N (2018). Bridging veins and veins of the brainstem in microvascular decompression surgery for trigeminal neuralgia and hemifacial spasm. Neurosurg Focus.

[33] Watanabe T, Igarashi T, Fukushima T, Yoshino A, Katayama Y (2013). Anatomical variation of superior petrosal vein and its management during surgery for cerebellopontine angle meningiomas. Acta Neurochir.

[34] Yang YM, Wang ZW, Cui Z, Jiang HZ, Sha C, Yuan QG, Xie HW, Wang DM (2017). Anatomy and management of superior petrosal vein in microvascular decompression for trigeminal neuralgia. Zhonghua Yi Xue Za Zhi.

[35] Yoshino N, Akimoto H, Yamada I, Nagaoka T, Tetsumura A, Kurabayashi T, Honda E, Nakamura S, Sasaki T (2003). Trigeminal neuralgia: evaluation of neuralgic manifestation and site of neurovascular compression with 3D CISS MR imaging and MR angiography. Radiology.

